# Integrating Technology Acceptance and Behavior Change Theories to Guide Technology Interventions

**DOI:** 10.1093/geroni/igaa057.1823

**Published:** 2020-12-16

**Authors:** Maurita Harris, Wendy Rogers

**Affiliations:** University of Illinois at Urbana-Champaign, Champaign, Illinois, United States

## Abstract

Technology interventions are commonly proposed as an effective means to support health self-management in older adults. For such interventions to be successful, we must identify individuals who are willing to adopt and adhere to these technologies. The general Technology Acceptance Model (TAM; Davis 1989) has been widely used to predict intentions to adopt technology in a variety of contexts. Likewise, the Theory of Planned Behavior (Azjen, 1991) has long been used to provide insights about health behaviors. These theories share three common stages: attitudes, behavior intentions, and acceptance. However, neither perspective provides insight into continued utilization of a technology tool (i.e., long-term adoption). Our goal is to integrate these models with the Transtheoretic Model of Behavior Change (Prochaska & Velicer, 1997) to provide insights that can help design technological interventions for older adults who want to change a health behavior and maintain that change over time.

